# Elinor Ostrom (1933–2012): Pioneer in the Interdisciplinary Science of Coupled Social-Ecological Systems

**DOI:** 10.1371/journal.pbio.1001405

**Published:** 2012-10-16

**Authors:** John M. Anderies, Marco A. Janssen

**Affiliations:** 1 School of Sustainability, Arizona State University, Tempe, Arizona, United States of America; 2 School of Human Evolution and Social Change, Arizona State University, Tempe, Arizona, United States of America

## Abstract

John Anderies and Marco Janssen pay tribute to an uncommon scholar.

**Figure pbio-1001405-g001:**
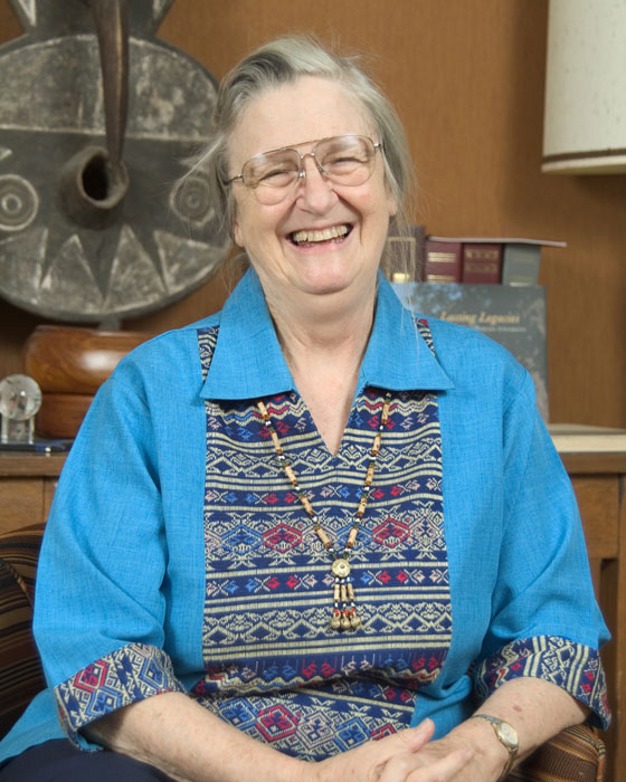
Elinor Ostrom. Image credit: Courtesy of Indiana University.

[Fig pbio-1001405-g001]Early exploration of environmental problems often cast them in terms of the inescapable tragedy of the commons. The “tragedy” refers to the subtle challenge associated with a fundamental disconnect between what is good for the individual and what is good for the group. The tragedy is a sad irony: in trying to serve their own self-interests, individuals end up hurting themselves—and the public good—in the long run. This “commons dilemma,” a core puzzle in the social sciences, is ubiquitous in both social and natural systems. Elinor Ostrom, who died June 12, 2012, at the age of 78, challenged the assumption that communities were incapable of developing their own strategies to avoid tragic outcomes.

The conventional wisdom when Ostrom began to shift her attention to this problem in the early 1980s was that the only way people could overcome the tragedy and utilize shared resources (groundwater, fisheries, and the atmosphere) wisely was either through strict, top-down government regulation or through privatization of the resource. Both approaches require significant public infrastructure, either to monitor and enforce regulations or to legitimize and enforce private property claims. However, as a result of her own observations and the field work of others, a body of evidence was accumulating that suggested groups are often able to successfully solve commons dilemmas without complex formal infrastructure for making, monitoring, and enforcing rules (e.g., formal legislative bodies, police forces, and scientific organizations) regarding the sustainable use of shared resources, in direct conflict with theoretical predictions. Ostrom bucked disciplinary research trends at the time and focused her energy on developing a new theory to explain what was happening in the field.

Through extensive fieldwork in many regions of the world, including Nepal, India, Mexico, Uganda, California, and Indiana, Ostrom began to uncover a robust pattern: self-organizing solutions to commons problems are not only possible, but in fact occur quite often. Rather than relying on formal government mechanisms or infrastructure, communities rely on a combination of informal norms, trust, and a small set of formal rules that the users themselves construct, monitor, and enforce using graduated sanctions. Ostrom's work developing a theoretical framework to study this effect earned her the 2009 Nobel Prize in economics, making her the first woman to be so honored. What is more, in developing this framework, Ostrom forged important linkages between the social and natural sciences. These linkages and the interdisciplinary methodology she helped develop to explore them are among her most important contributions—contributions that biologists would do well to learn from.

Ostrom did not always work across disciplines, but given the time period in which she worked, she was likely not able to. Although she focused on the management of shared groundwater resources in Southern California in her PhD thesis (finished in 1965), her work shifted away from natural resource problems during her first 15 years on the political science faculty at Indiana University. In that period she focused on disciplinary political science questions associated with police forces in US cities, and what types of organizational forms led to the most effective policing. After this period, beginning in the early 1980s, Ostrom began to develop a more theoretical understanding of the rules and norms that people use to organize themselves, influenced by a sabbatical spent in Germany with Nobel Laureate economist Reinhard Selten. This effort resulted in the creation of the Institutional Analysis and Development (IAD) framework—a systemic method for comparative analysis of case studies. This framework was strongly resisted at first, especially within political science, but today it is used widely in the social sciences.

During the mid-1980s Ostrom returned to the study of the commons and put the IAD to work. At the time, it was becoming increasingly recognized that the experience of groups managing shared resources clashed with the conventional view that the resource would inevitably be overexploited. Ostrom's ideas were critical in providing a theoretical foundation for these observations. She conducted field research around the world and synthesized the case studies of others, resulting in the compilation of hundreds of case studies of self-organizing governance, from the lobster fisheries of Maine to the irrigation systems of Nepal. Using ideas from the IAD framework to systematically compare conditions across these cases, she identified eight design principles that characterized successful self-governance strategies, including having monitors who are accountable to the users of a resource, and inexpensive (in terms of time and effort) mechanisms for conflict resolution. Those principles have held up to the test of time and led to her now classic 1990 book, *Governing the Commons*.

Ostrom's work in *Governing the Commons* pushed intellectual boundaries, but she was still not satisfied. She wanted to place it on a firmer theoretical foundation and did so by extending her work in two key areas. The first was experimental economics, pioneered by Charles Plott and Vernon Smith (winner of the Nobel Prize in economics for his work in this area) in the 1970s. Experimental economics involves creating games that mimic economic situations including markets, individual and collective decision making, and bargaining and observing the choices people make when they play such games in a controlled environment. Ostrom's work in experimental economics was aimed at developing a more robust understanding of how people actually behave (based on the decisions they make) in commons situations. Beginning in the late 1980s, Ostrom designed a series of controlled experiments to test her insights. Over and over again, Ostrom and her colleagues replicated principles of self-governance in the lab. She showed that without communication, the theoretical predictions from economics hold—people will not cooperate. Even with informal communication, however, she showed that people can and do solve commons dilemmas easily. These experiments showed that humans might be better labelled as *Homo cooperaticus*, who are naturally willing to work together but may quit if they are suckered, rather than *Homo economicus*, who are rational, narrowly self-interested, and never willing to cooperate.

Ostrom's comparative case study analysis and experimental work were both strongly rooted in the social sciences. The last phase in Ostrom's work focused on developing tools and ideas to understand how subtle *micro contextual* variables related to social, physical, hydrological, and ecological context affect the capacity of groups to solve collective action problems. Specifically, “attributes of the physical world” was a core element in the IAD framework that Ostrom had not yet fully explored. She understood well that actors' choices impacted the physical world, which subsequently conditioned future choices and social organization. Thus, she needed a *dynamic*, system-level approach to study social-ecological systems (SESs). For this, Ostrom first turned to ideas from theoretical ecology and resilience, then to complex adaptive systems (CASs) and robustness. Reslience, rooted in ecology, is a loosely organized cluster of concepts relating to the interplay of transformation and persistence of non-linear dynamical systems. Resilience emphasizes that ecological, and more broadly, SESs, are composed of multiple elements that interact across scales and levels of organization. These interactions generate regimes that SESs occupy, and slow changes in variables (e.g., phosphorus loading in lakes) that structure those regimes can induce rapid regime shifts (flips in lakes from oligotrophic to eutrophic states). Likewise, CASs are systems composed of multiple elements that interact locally that generate system-level dynamics that are often emergent and unpredictable. Ostrom saw a clear parallel between these concepts and institutions. Institutions are rules that structure how individual elements in the system (people, animal species) interact, which, in turn, structures how the SES behaves at the macro level (e.g., whether it is sustainable or not). As a result, she worked very hard to extend the IAD framework to create a more prominent role for natural science knowledge and eventually developed a general framework for analyzing the sustainability of SESs. The framework, known as the “SES Framework,” conceptualizes SESs in terms of four core interacting subsystems—the resource system, resource units, governance system, and resource users—and provides a methodology to systematically unpack these elements in terms of second-level variables under first-level core subsystems, and so on. The SES Framework, detailed in *Science* (2009, 325: 419–422) and *PNAS* (2007, 104: 15181–15187), is increasingly used today to examine case studies involving everything from single watersheds to global climate change.

Ostrom's legacy will undoubtedly impact scholarship in diverse ways. But one of the most important lessons she left for scholars across the natural and social sciences is that, although trained as a social scientist, Ostrom was willing to attack problems with a wide range of methods from a variety disciplines and embrace emerging, challenging concepts from other disciplines, such as complex adaptive systems, to study the questions that interested her. She strongly believed that the research question should drive the methods employed, not the discipline of the person asking the question. Ostrom did not just play lip service to this high ideal, she lived it and suffered severe criticism from her own discipline.

Over her career, Ostrom was a tireless advocate of interdisciplinary research and, with her husband Vincent Ostrom, founded the Workshop in Political Theory and Policy Analysis at Indiana University. The term “workshop” is deliberate, intended to emphasize science as *practice* and scientists as craftspersons who become masters of their craft. The Workshop is a place that has brought, and will continue to bring together, scholars from various stripes—ecologists, economists, anthropologists, hydrologists, climatologists, sociologists, computer scientists, mathematicians, and geographers—to tackle the most pressing contemporary problems involving the dynamics of SESs from local to global scales. Thanks to Elinor Ostrom, a woman with uncommon dedication and vision, we now have an intellectual framework and a practical model for working together across disciplines to confront these problems with the full range of methods and expertise we will surely need.

